# Individual, societal and clinical burden of cardiovascular disease: health technology assessment to guide new diagnostics and treatment strategies

**DOI:** 10.1007/s12471-023-01787-y

**Published:** 2023-05-09

**Authors:** Jolanda van der Velden

**Affiliations:** 1 Department of Physiology, Amsterdam University Medical Centres, location Free University Amsterdam, Amsterdam, The Netherlands; 2 Amsterdam Cardiovascular Sciences, Amsterdam University Medical Centres, location Free University Medical Centre, Amsterdam, The Netherlands

Based on data from the World Health Organization and World Bank in 2018, mortality rates caused by ischaemic heart disease in the Netherlands are amongst the lowest in Europe, at a relatively high health expenditure [1]. While age-standardised mortality from all-cause cardiovascular disease (CVD), ischaemic heart disease and stroke decreased in past years in both males and females [2], morbidity and associated disability rates will increase in the ageing population. In addition, unhealthy food combined with a sedentary lifestyle will increase CVD incidence in younger people. Moreover, increased knowledge of genetics shows that a relatively large patient group suffers from inherited CVD at young age.

The increase in cardiovascular morbidity represents an enormous challenge for our society and healthcare system. In 2019, healthcare costs amounted to 6.8 milliard euros, with the highest costs for men and a peak between 70 and 74 years of age [3]. Parallel to increasing healthcare costs, new innovations for the diagnosis and treatment of CVD are ready to enter the clinic. A major question is whether all newly developed diagnostic tests, cardiovascular imaging and interventions are more cost-effective than current clinical care and whether new treatments improve health-related quality of life (QoL) and reduce societal burden. Thus, to even further optimise patient care, including optimisation of diagnosis and therapy, it is essential to define the current costs of CVD care and define QoL and costs related to disability.

In this special issue of the *Netherlands Heart Journal*, Wiethoff and colleagues offer an introduction to health technology assessment (HTA) and different types of economic evaluations [4]. We invite you to go to the online self-learning course, which provides explanations of the different aspects of economic evaluations ranging from costs and outcomes to the various methods in an easily accessible manner.

While global healthcare costs for CVD are known [3], exact knowledge of disease-specific costs for care and data on disease-specific QoL are lacking. Due to its increasing prevalence, it is assumed that inherited forms of CVD have a high impact on patients, their family members and their surroundings. Current screening, diagnosis and treatment of inherited cardiomyopathy are costly and time-consuming and follow a ‘one size fits all’ approach, thereby ignoring the variation in the cardiomyopathy population. Current internationally used diagnostic methods to exclude a cardiomyopathy phenotype in family members are based on yearly or biannual cardiology visits that include imaging but do not use artificial intelligence. Novel methods that use machine learning-based analysis of electrocardiograms and imaging and development of easy-to-use risk scores to predict the onset and disease progression in cardiomyopathy have the potential to decrease the psychological impact on patients and relatives and decrease healthcare costs.

Early HTA can give insight into the potential value of important decision problems, i.e. which screening strategies, diagnostic strategies and treatment strategies to study, relating to the management of subpopulations of individuals with inherited cardiomyopathy. A systematic literature search indeed illustrated that there is limited current knowledge of the societal and economic burden of the most common forms of inherited cardiac disease: hypertrophic cardiomyopathy (HCM) and dilated cardiomyopathy [5]. To increase knowledge of QoL and societal costs of HCM in the Netherlands, a prospective burden of disease study in HCM patients and preclinical genotype-positive individuals has been designed [6]. The results of such early HTAs will not only give insight into the potential cost-effectiveness of different diagnostics and interventions, but will also guide the future research agenda on inherited cardiomyopathies, i.e. to assess the effectiveness and cost-effectiveness of novel interventions.

While HTA in inherited cardiac disease is at an early stage, studies of HTA in the Netherlands on complications and health care costs of implantable cardioverter defibrillators (ICDs) and on management of acute ischaemic stroke are herein presented. ICD implantation is standard of care for the primary prevention of sudden cardiac arrest, but Van Barreveld and colleagues show that complication-related costs should be taken into account when considering the overall cost-effectiveness of device therapy [7]. Van den Berg et al. show that endovascular treatment for patients with acute ischaemic stroke was not only successful in terms of QoL, but also positive from a cost perspective [8]. Their economic impact analysis performed as part of the MR CLEAN trial indicated that the intervention was increasingly cost-saving for national healthcare.

This issue thus shows the potential of HTA to quantify the cost-effectiveness of current diagnostics and clinical care from both a patient and societal perspective and the impact of novel therapies. Future research needs to include quality-adjusted life years and a broader range of costs to provide an information base for optimising care for affected patients.
